# N-terminal pro-brain natriuretic peptide levels had an independent and added ability in the evaluation of all-cause mortality in older Chinese patients with atrial fibrillation

**DOI:** 10.1186/s12877-019-1051-0

**Published:** 2019-02-28

**Authors:** Shihui Fu, Jie Jiao, Yi Guo, Bing Zhu, Leiming Luo

**Affiliations:** 1 Department of Geriatric Cardiology, Chinese People’s Liberation Army General Hospital, Medical School of Chinese People’s Liberation Army, Beijing, 100853 China; 2 Department of Cardiology, Hainan Hospital of Chinese People’s Liberation Army General Hospital, Sanya, China; 3 Intensive Care Unit, Hainan Hospital of Chinese People’s Liberation Army General Hospital, Sanya, China

**Keywords:** Atrial fibrillation, Older Chinese patients, N-terminal pro-brain natriuretic peptide, All-cause mortality

## Abstract

**Background:**

Atrial fibrillation (AF) is the most common arrhythmia and has increased prevalence in older patients, leading to poor prognosis for these patients. There is a need for a biomarker or a model of prognostic evaluation in older patients with AF, especially in China. CHADS_2_ and CHA_2_DS_2_VASc scores have been applied to evaluate their prognosis in patients with AF. This analysis was designed to examine whether N-terminal pro-brain natriuretic peptide (NT-proBNP) levels significantly improved the evaluation of all-cause mortality in older Chinese patients with AF when added to CHADS_2_ and CHA_2_DS_2_VASc scores.

**Methods:**

There were 219 older patients with AF, and follow-up was 100% complete over an average of 1.11 years. Cox regression analysis was applied to determine the variables independently associated with all-cause mortality.

**Results:**

Median age was 85 years, and all-cause mortality was 24.2% (53 patients). Plasma NT-proBNP levels were significantly associated with all-cause mortality in univariate [hazard ratio (HR): 1.842; 95% confidence interval (CI): 1.530–2.218] and multivariate (HR: 1.377; 95% CI: 1.063–1.785) Cox regression analyses and had significantly higher c-statistic (0.771; 95% CI: 0.698–0.845) than CHADS_2_ (0.639; 95% CI: 0.552–0.726) and CHA_2_DS_2_VASc (0.633; 95% CI: 0.546–0.720) scores (*P* < 0.05 for all). The addition of NT-proBNP levels to CHADS_2_ (0.783; 95% CI: 0.713–0.854) and CHA_2_DS_2_VASc (0.775; 95% CI: 0.704–0.846) scores significantly increased their c-statistics (*P* < 0.001 for all). Model based on NT-proBNP levels including age, hemoglobin, fasting blood glucose, glomerular filtration rate and NT-proBNP levels had a significantly higher c-statistic (0.890; 95% CI: 0.841–0.938) than CHADS_2_ and CHA_2_DS_2_VASc scores (P < 0.001 for all). Model based on NT-proBNP levels had significantly higher c-statistic than the addition of NT-proBNP levels to CHADS_2_ and CHA_2_DS_2_VASc scores (*P* < 0.05).

**Conclusion:**

NT-proBNP levels were an independent biomarker associated with an increased all-cause mortality in older Chinese patients with AF, and had an independent and added ability to evaluate their all-cause mortality compared with CHADS_2_ and CHA_2_DS_2_VASc scores.

## Background

Atrial fibrillation (AF) is the most common arrhythmia and has an increased prevalence in older patients, leading to poor prognosis for these patients [1, 2]. There is a need for a biomarker or a model of prognostic evaluation in older patients with AF, especially in China [3]. CHADS_2_ and CHA_2_DS_2_VASc scores have been applied to evaluate their prognosis [4]. CHADS_2_ score includes age, congestive heart failure, hypertension, diabetes mellitus, and prior stroke or transient ischemic attack (TIA). CHA_2_DS_2_VASc score includes age, female gender, congestive heart failure, hypertension, vascular diseases, diabetes mellitus, and prior stroke or TIA. But as the commonly applied scores to evaluate thrombotic risk, they are in need of improvement for prognostic evaluation [4]. N-terminal pro-brain natriuretic peptide (NT-proBNP) is a stable 76-amino acid N-terminal segment of pro-brain natriuretic peptide (pro-BNP), and significantly related to poor prognosis in patients with different cardiovascular diseases, including coronary artery disease and heart failure [5]. Moreover, NT-proBNP levels can also be applied to improve the roles of traditional scores, such as Seattle Heart Failure Score (SHFS), in prognostic evaluation of patients with these cardiovascular diseases [6]. NT-proBNP levels have been found to be associated with heart failure mortality in the Apixaban for Reduction in Stroke and Other Thromboembolic Events in Atrial Fibrillation (ARISTOTLE) trial, which consists of 18,201 patients with AF treated with apixaban or warfarin [7]. Meanwhile, NT-proBNP levels have been found to be a significant predictor of all-cause mortality in the Randomized Evaluation of Long-Term Anticoagulation Therapy (RE-LY) trial, which consists of 18,113 patients with AF treated with dabigatran or warfarin [8]. The addition of NT-proBNP levels to CHADS_2_ and CHA_2_DS_2_VASc scores or a model based on NT-proBNP levels may provide better methods to predict prognosis in patients with AF. To our knowledge, no study has assessed whether NT-proBNP levels improve prognostic evaluation of CHADS_2_ and CHA_2_DS_2_VASc scores, especially in older Chinese patients. Age and race can also have significant effects on the prognostic evaluation of patients with AF, and further studies are needed to assess the effects of adding NT-proBNP levels to CHADS_2_ and CHA_2_DS_2_VASc scores on the prognostic evaluation of older Chinese patients with AF [9]. The aim of the current analysis was to examine whether NT-proBNP levels significantly improved the evaluation of all-cause mortality in older Chinese patients with AF when added to CHADS_2_ and CHA_2_DS_2_VASc scores.

## Methods

### Study patients

The current analysis included 219 patients (> 60 years) with AF, as determined from histories, symptoms (palpitation), signs (arrhythmia) and electrocardiograph, as established by the chief physicians according to the American College of Cardiology (ACC)/American Heart Association (AHA)/European Society of Cardiology (ESC) guidelines for AF. The Chinese People’s Liberation Army General Hospital was their designated hospital and had their integrated long-term medical and final death records, which made it easier for us to follow up these patients effectively and judge end point accurately. All these patients were hospitalized at the moment of inclusion.

### Biochemical assays

Blood samples were drawn in a fasting state and assayed by qualified technicians without the knowledge of the clinical data in a standardized procedure at the central laboratory in the Department of Biochemistry, Chinese People’s Liberation Army General Hospital. Serum NT-proBNP levels were measured with the NT-proBNP Flex Reagent Cartridge, produced by Siemens Healthcare Diagnostics, on the Dimension RXL Max [analytical measuring range (AMR): 10–30,000 pg/mL]. Levels of fasting blood glucose (FBG), cholesterol, high-density lipoprotein cholesterol (HDL-c) and low-density lipoprotein cholesterol (LDL-c) were quantified with the Roche enzymatic assays (Roche Products Ltd., Basel, Switzerland) on the Roche autoanalyzer (Roche Products Ltd., Basel, Switzerland). Serum creatinine levels were measured with the enzymatic assay. Glomerular filtration rate (GFR) was calculated with the Chinese modified Modification of Diet in Renal Disease (MDRD) equation: 175 × plasma creatinine ^− 1.234^ × age ^− 0.179^ × 0.79 (if female) [10].

### Diagnostic definitions

Body mass index (BMI) was defined as weight in kilograms divided by the square of height in meters. Congestive heart failure was defined based on long-term symptoms (dyspnea and/or fatigue), signs (edema and/or pulmonary rales), and abnormalities of cardiac structure or function, as established by the chief physicians according to ESC guideline for congestive heart failure. Standard echocardiography was conducted by experts, and left ventricular ejection fraction was evaluated based on Simpson’s method [11]. Paroxysmal AF was self-terminating, in most cases within 48 h. and in some cases up to 7 days. AF episodes that were cardioverted within 7 days should be considered paroxysmal AF. Persistent AF last longer than 7 days, including episodes that were terminated by cardioversion, either with drugs or by direct current cardioversion, after 7 days or more. Permanent AF was diagnosed when AF was accepted by the patient (and physician), and rhythm control interventions were not pursued in these patients. Hypertension was defined if patients had mean systolic blood pressure ≥ 140 mmHg, mean diastolic blood pressure ≥ 90 mmHg or antihypertensive drugs. Five separate measurements of systolic and diastolic blood pressures were conducted to obtain mean systolic and diastolic blood pressures. Blood pressures were measured on two consecutive mornings, in the first afternoon and on two consecutive nights. Diabetes mellitus was defined if patients had FBG ≥ 7.0 mmol/L or oral hypoglycemic drugs/insulins. Vascular diseases were defined as myocardial infarction and peripheral artery disease. Myocardial infarction was determined from histories, symptoms (typical angina), cardiac markers, and cardiac examinations, such as electrocardiogram, echocardiography, radionuclide imaging, computed tomography and coronary angiography, as established by the chief physicians according to ACC/AHA guideline for myocardial infarction. Peripheral artery disease was determined from histories, symptoms (pain), signs (claudication), and examinations, such as echocardiography and angiography, as established by the chief physicians according to European Society for Vascular Surgery (ESVS) guideline. Stroke was defined if patients had new and sudden focal neurological deficit caused by a presumed cerebrovascular reason lasting > 24 h, after eliminating other identifiable reasons including tumor or seizure. TIA was defined as if patients had symptoms lasting < 24 h.

### Current scores

CHADS_2_ score included the following factors: age (one point: ≥ 75 years), congestive heart failure (one point), hypertension (one point), diabetes mellitus (one point), and prior stroke or TIA (two points). CHA_2_DS_2_VASc score included the following factors: age (two points: ≥ 75 years; one point: 65–74 years; zero point: ≤ 64 years), female gender (one point), congestive heart failure (one point), hypertension (one point), vascular diseases (one point), diabetes mellitus (one point), and prior stroke or TIA (two points). There were 173 (79.0%) patients receiving antiplatelet drugs, and 46 patients (21.0%) receiving anticoagulants.

### Prognostic evaluation

Due to the priority of all-cause mortality in the outcome studies, as well as an increased risk of multiple organ failure in older patients, the primary terminal point in the current analysis was all-cause mortality. Multiple organ failure was the main cause of death in these older patients. Follow-up was 100% complete over an average of 1.11 years (406 days; median: 313 days; interquartile range: 199–532 days). Follow-up time for all patients included follow-up time of patients that died. Follow-up data were tracked directly from medical records and telephone interviews. Death was determined from death records, a legal document including the time, site and other information.

### Statistical analyses

Continuous variables were summarized as mean and standard deviation (normal distribution) or median and interquartile range (skewed distribution). Coefficient of variation was also applied as a descriptive method. Categorical variables were summarized as number and percentage. NT-proBNP levels were logarithmically transformed to meet the multivariate normality assumption. Univariate Cox regression analysis was applied to evaluate the relationship between variables and all-cause mortality, and covariates with *P* < 0.10 were taken into multivariate Cox regression analysis with the Enter method to determine the variables independently associated with all-cause mortality. It is generally believed that co-linearity has no obvious effect on the regression analyses if tolerances are ≥0.1 and variance inflation factors are ≤5–10. All tolerances were > 0.5 and all variance inflation factors were < 1.8 in the current regression analysis. Thus, co-linearity had no obvious effect on the current regression analysis. Receiver-operating characteristic curve was plotted and area under the curve (c-statistics) represented the ability of NT-proBNP levels in evaluating all-cause mortality. The Z test was applied to compare the c-statistics so as to evaluate the added abilities of NT-proBNP levels to current scores. Statistical analyses were conducted with Statistical Package for Social Sciences version 17 (SPSS Inc., Chicago, IL, USA) and MedCalc 9.6 for Windows (MedCalc Software bvba, Mariakerke, Belgium). Two-sided *P* values were regarded as significant if they were less than 0.05.

## Results

**Fig. 1:**
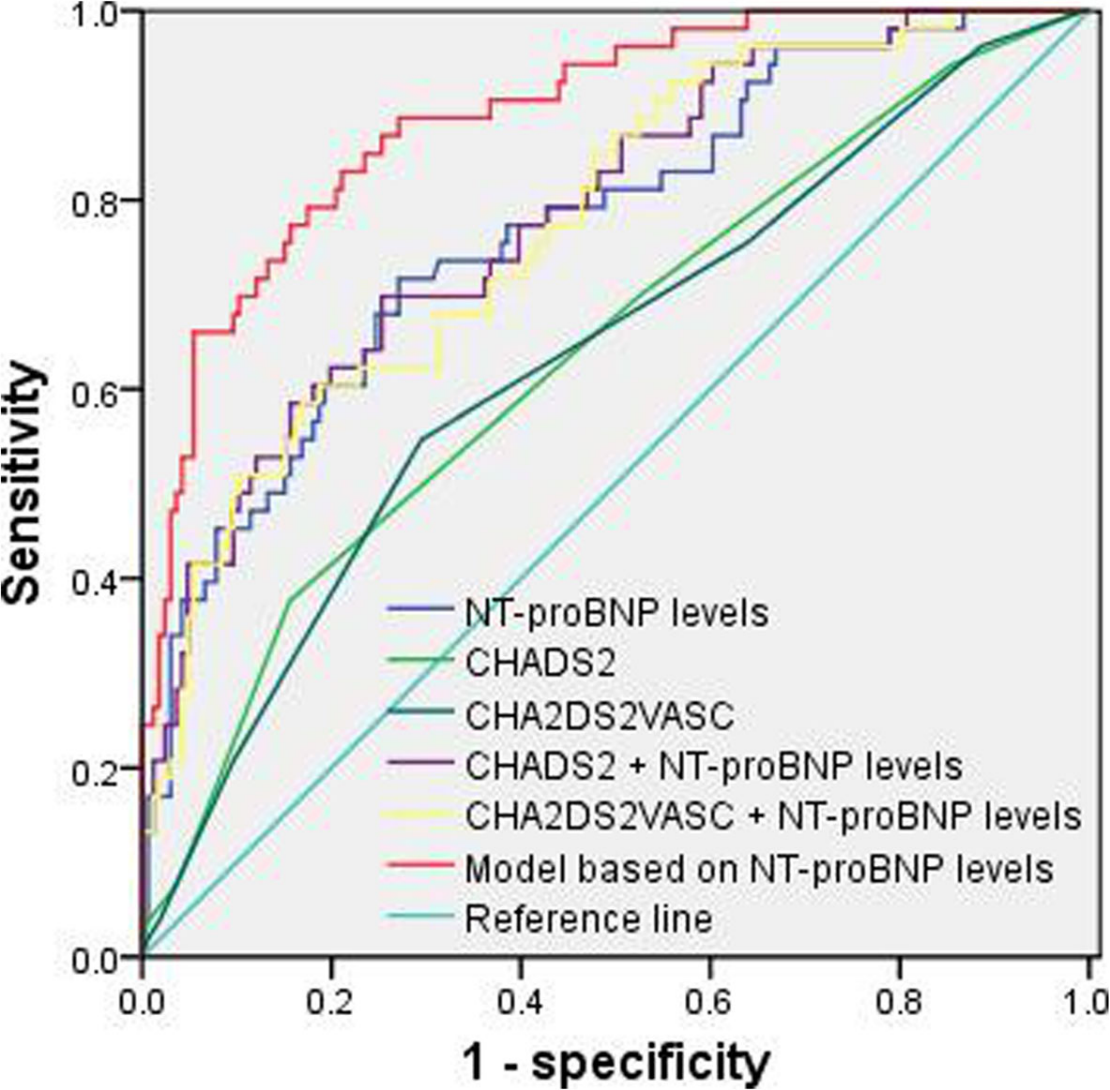
Comparison of c-statistics between NT-proBNP levels, CHADS_2_, CHA_2_DS_2_VASc, and model based on NT-proBNP levels. Abbreviations: NT-proBNP: N-terminal pro-brain natriuretic peptide

Among these patients with AF, 128 patients (58.4%) had paroxysmal AF, 44 patients (20.1%) had persistent AF, and 47 patients (21.5%) had permanent AF. Patient features are reported in Table 1. These patients had a median age of 86 years, a median CHADS_2_ score of 3.0, a median CHA_2_DS_2_VASc score of 4.0 and a median NT-proBNP level of 1333.0 pg/mL (coefficient of variation: 2.02). All-cause mortality was 24.2% (53 patients) during the follow-up. NT-proBNP levels were significantly associated with all-cause mortality in univariate Cox regression analysis [hazard ratio (HR): 1.84; 95% confidence interval (CI): 1.53–2.22; *P* < 0.001; Table 1) and had significantly higher c-statistic (0.77; 95% CI: 0.70–0.85) than CHADS_2_ (0.64; 95% CI: 0.55–0.73); *P* = 0.013) and CHA_2_DS_2_VASc (0.63; 95% CI: 0.55–0.72; *P* = 0.009) scores (Table 2; Fig. 1). The addition of NT-proBNP levels to CHADS_2_ (0.78; 95% CI: 0.71–0.85; *P* < 0.001) and CHA_2_DS_2_VASc (0.78; 95% CI: 0.70–0.85; *P* < 0.001) scores significantly increased their c-statistics. Age, hemoglobin, FBG, GFR and NT-proBNP levels (HR: 1.38; 95% CI: 1.06–1.79) were significantly associated with all-cause mortality in multivariate Cox regression analysis (*P* < 0.05; Table 3). Model based on NT-proBNP levels including age, hemoglobin, FBG, GFR and NT-proBNP had a significantly higher c-statistic (0.890; 95% CI: 0.841–0.938) than CHADS_2_ (P < 0.001) and CHA_2_DS_2_VASc (*P* < 0.001). Model based on NT-proBNP levels including age, hemoglobin, FBG, GFR and NT-proBNP levels had significantly higher c-statistic than the addition of NT-proBNP levels to CHADS_2_ (*P* = 0.003) and CHA_2_DS_2_VASc (*P* = 0.001) scores.

**Table 1:** Patient features and their effects on all-cause mortality

Variables	Descriptions	HR^a^	95 CI^a^	*P* ^a^
Demographics				
Age (year) ^b^	86(82–90)	1.08	1.03–1.14	0.003
Males (%)	187(85.4)	0.83	0.37–1.84	0.642
BMI (kg/m^2^) ^c^	24.1(3.8)	0.95	0.88–1.03	0.189
Diseases				
Congestive heart failure/ventricular function< 40% (%)	102(46.6)	2.45	1.39–4.33	0.002
Hypertension (%)	170(77.6)	1.30	0.63–2.66	0.476
Diabetes mellitus (%)	78(35.6)	1.95	1.14–3.34	0.015
Stroke/TIA (%)	20(9.1)	0.81	0.29–2.25	0.690
Vascular diseases (%)	51(23.3)	1.22	0.66–2.24	0.526
Clinical presentation				
Heart rate (bpm) ^b^	74(64–84)	1.02	1.01–1.03	0.003
SBP (mmHg) ^b^	132(123–141)	1.00	0.98–1.02	0.666
DBP (mmHg) ^b^	70(64–76)	0.98	0.95–1.01	0.223
Laboratory results				
Hemoglobin (g/L) ^b^	123.0(107.0–136.0)	0.96	0.95–0.97	< 0.001
Plasma albumin (g/L) ^c^	37.7(4.2)	0.82	0.78–0.87	< 0.001
FBG (mmol/L) ^b^	5.5(4.9–6.3)	1.17	1.10–1.25	< 0.001
Cholesterol (mmol/L) ^b^	3.7(3.2–4.3)	0.88	0.64–1.21	0.434
HDL-c (mmol/L) ^b^	1.0(0.9–1.2)	0.13	0.05–0.32	< 0.001
LDL-c (mmol/L) ^b^	2.1(1.6–2.5)	0.87	0.59–1.28	0.489
Uric acid (mmol/L) ^b^	352.4(270.1–441.1)	1.00	1.00–1.01	0.021
GFR (ml/min/1.73 m^2^) ^b^	65.8(52.6–78.6)	0.97	0.96–0.98	< 0.001
NT-proBNP (pg/mL) ^b^	1333.0(463.4–3101.8)	1.84	1.53–2.22	< 0.001

Notes: ^a^univariate Cox regression analyses; ^b^median (interquartile range); ^c^mean (standard deviation)

Abbreviations: *HR* hazard ratio, *CI* confidential interval, *BMI* body mass index, *TIA* transient ischemic attack, *SBP* systolic blood pressure, *DBP* diastolic blood pressure, *FBG* fasting blood glucose, *HDL-C* high-density lipoprotein cholesterol, *LDL-C* low-density lipoprotein cholesterol, *GFR* glomerular filtration rate, *NT-proBNP* N-terminal pro-brain natriuretic peptide

**Table 2:** Comparison of NT-proBNP levels, CHADS_2,_ CHA_2_DS_2_VAS_C_, and model based on NT-proBNP levels in the evaluation of all-cause mortality

	C-statistic	95% CI	*P*	Z	*P*
NT-proBNP levels	0.77	0.70–0.85	< 0.001		
CHADS_2_	0.64	0.55–0.73	0.002	2.47	0.013^a^
CHA_2_DS_2_VAS_C_	0.63	0.55–0.72	0.045	2.62	0.009^b^
CHADS_2_ + NT-proBNP levels	0.78	0.71–0.85	< 0.001	3.63	< 0.001^c^
CHA_2_DS_2_VAS_C_ + NT-proBNP levels	0.78	0.70–0.85	< 0.001	3.31	< 0.001^d^
Model based on NT-proBNP levels	0.89	0.84–0.94	< 0.001	5.25	< 0.001^e^
				5.31	< 0.001^f^
				3.02	0.003^g^
				3.30	0.001^h^

Notes: ^a^*P* value was drawn from comparison between CHADS_2_ and NT-proBNP levels; ^b^P value was drawn from comparison between CHA_2_DS_2_VAS_C_ and NT-proBNP levels; ^c^P value was drawn from comparison between CHADS_2_ + NT-proBNP levels and CHADS_2_; ^d^P value was drawn from comparison between CHA_2_DS_2_VAS_C_ + NT-proBNP levels and CHA_2_DS_2_VAS_C_; ^e^P value was drawn from comparison of model based on NT-proBNP levels with CHADS_2_; ^f^P value was drawn from comparison of model based on NT-proBNP levels with CHA_2_DS_2_VAS_C;_^g^P value was drawn from comparison of model based on NT-proBNP levels with CHA_2_DS_2_ + NT-proBNP levels; ^h^P value was drawn from comparison of model based on NT-proBNP levels with CHA_2_DS_2_VAS_C_ + NT-proBNP levels

Abbreviations: *NT-proBNP* N-terminal pro-brain natriuretic peptide, *CI* confidence interval

**Table 3:** Factors independently associated with all-cause mortality (model based on NT-proBNP levels)

Factors^a^	HR^a^	95% CI^a^	*P* ^a^
Age (year)	1.07	1.01–1.13	0.016
Hemoglobin (g/L)	0.98	0.97–1.00	0.040
FBG (mmol/L)	1.16	1.05–1.27	0.003
GFR (ml/min/1.73 m^2^)	0.98	0.97–1.00	0.008
NT-proBNP (pg/mL)	1.38	1.06–1.79	0.016

Notes: ^a^multivariate Cox regression analysis

Abbreviations: *HR* hazard ratio, *CI* confidence interval, *FBG* fasting blood glucose, *GFR* glomerular filtration rate, *NT-proBNP* N-terminal pro-brain natriuretic peptide

## Discussion

Prevalent AF results in severe disability or death, and consequently it is essential to evaluate prognosis in patients with AF [1, 2]. At present, no biomarker or method is satisfactory in evaluating prognosis in patients with AF, and CHADS_2_ and CHA_2_DS_2_VASc scores have been applied to evaluating prognosis in these patients [3, 4]. NT-proBNP levels have been suggested to correlated significantly with poor prognosis of patients with different cardiovascular diseases including coronary artery disease and heart failure [5]. In addition, other studies have also supported the incremental abilities of NT-proBNP levels to traditional scores, such as SHFS, in prognostic evaluation of patients with these cardiovascular diseases [6]. In patients with AF, NT-proBNP levels have recently been found to be associated with heart failure mortality in the ARISTOTLE trial [7]. Meanwhile, the RE-LY trial has demonstrated that NT-proBNP levels were a significant predictor of all-cause mortality [8]. However, to our knowledge, no study has assessed whether NT-proBNP levels improve the evaluation of all-cause mortality in older Chinese patients compared with CHADS_2_ and CHA_2_DS_2_VASc scores [9]. The current analysis confirmed that elevated NT-proBNP levels were independently associated with an increased all-cause mortality, and provided added information concerning the evaluation of all-cause mortality in patients with AF even better than CHADS_2_ and CHA_2_DS_2_VASc scores.

NT-proBNP levels are increased in cardiomyocytes in response to atrial strain and dilation, and have been found to correlate with poor prognosis of patients with AF [12]. NT-proBNP levels are elevated in patients with HF, and have been shown to be a risk factor for poor prognosis of patients with HF [13]. Elevated NT-proBNP levels in patients without HF may reflect a cumulative exposure of risk factors for myocardial systolic or diastolic subclinical dysfunction, which may result in increased filling pressure and poor prognosis of patients with AF [14]. Meanwhile, renal dysfunction has been regarded as a risk factor for poor prognosis of patients with AF, and slight decline in renal function can contribute to poor prognosis of patients with AF [15]. The current analysis suggested that as a sensitive biomarker of cardiac and renal dysfunction, NT-proBNP levels had an elevated levels independently associated with an increased all-cause mortality, and performed better than CHADS_2_ and CHA_2_DS_2_VASc scores in the evaluation of all-cause mortality in older Chinese patients with AF.

The current study had strengths and limitations. Firstly, there has been no widely-accepted model based on a biomarker reflecting cardiac and renal dysfunction in older patients with AF, and there is a need to build a model based on NT-proBNP levels [16]. The current analysis not only provided evidence of an independent and added ability of NT-proBNP levels in the evaluation of all-cause mortality, but also constructed a model based on NT-proBNP levels to evaluate all-cause mortality in older Chinese patients with AF. Model based on NT-proBNP levels could help clinical doctors to identify patients with high risk and poor prognosis, and provide comprehensive management of high quality for them to achieve prognostic improvement. However, it should be further validated in large-scale studies and different older populations, especially through the methods of net reclassification improvement and integrated discrimination improvement. Secondly, the current analysis selected the variables independently associated with all-cause mortality to form a model based on NT-proBNP levels. Hazard ratios of different variables obtained in the current analysis may be applied to develop a formula to make a prognostic evaluation. However, due to a lack of validation from large-scale studies, it may not be possible to determine this formula at present. Thirdly, the current analysis included all-cause mortality as the primary terminal point, but not did not take the causes of death into consideration and represent the number of events. However, with an increased risk of multiple organ failure, it is very difficult to determine the causes of death and the number of events in older patients. Moreover, all-cause mortality has its priority in the outcome studies, so it was continuously emphasized in the current analysis.

## Conclusion

The current analysis demonstrated that NT-proBNP levels were an independent biomarker associated with an increased all-cause mortality in older Chinese patients with AF, and had an independent and added ability to evaluate their all-cause mortality compared with CHADS_2_ and CHA_2_DS_2_VASc scores.

## Abbreviations

ACC: American College of Cardiology
AF: Atrial fibrillation
AHA: American Heart Association
AMR: Analytical measuring range
ARISTOTLE: Apixaban for Reduction in Stroke and Other Thromboembolic Events in Atrial Fibrillation
BMI: Body mass index
CI: Confidence interval
ESC: European Society of Cardiology
ESVS: European Society for Vascular Surgery
FBG: Fasting blood glucose
GFR: Glomerular filtration rate;
HDL-c: High-density lipoprotein cholesterol
HR: Hazard ratio
LDL-c: Low-density lipoprotein cholesterol
NT-proBNP: N-terminal pro-brain natriuretic peptide
RE-LY: Randomized Evaluation of Long-Term Anticoagulation Therapy
SHFS: Seattle Heart Failure Score
SPSS: Statistical Package for Social Sciences
TIA: Transient ischemic attack
